# Host Genetic Background Strongly Affects Pulmonary microRNA Expression before and during Influenza A Virus Infection

**DOI:** 10.3389/fimmu.2017.00246

**Published:** 2017-03-21

**Authors:** Matthias Preusse, Klaus Schughart, Frank Pessler

**Affiliations:** ^1^Institute for Experimental Infection Research, TWINCORE Center for Experimental and Clinical Infection Research, Hannover, Germany; ^2^Helmholtz Centre for Infection Research, Braunschweig, Germany; ^3^Department of Infection Genetics, Helmholtz Centre for Infection Research, Braunschweig, Germany; ^4^University of Veterinary Medicine Hannover, Hannover, Germany; ^5^Department of Microbiology, Immunology and Biochemistry, University of Tennessee Health Science Centre, Memphis, TN, USA; ^6^Centre for Individualised Infection Medicine, Hannover, Germany

**Keywords:** biomarkers, genetics, infection, influenza virus, lung, miRNA, mouse, susceptibility

## Abstract

**Background:**

Expression of host microRNAs (miRNAs) changes markedly during influenza A virus (IAV) infection of natural and adaptive hosts, but their role in genetically determined host susceptibility to IAV infection has not been explored. We, therefore, compared pulmonary miRNA expression during IAV infection in two inbred mouse strains with differential susceptibility to IAV infection.

**Results:**

miRNA expression profiles were determined in lungs of the more susceptible strain DBA/2J and the less susceptible strain C57BL/6J within 120 h post infection (hpi) with IAV (H1N1) PR8. Even the miRNomes of uninfected lungs differed substantially between the two strains. After a period of relative quiescence, major miRNome reprogramming was detected in both strains by 48 hpi and increased through 120 hpi. Distinct groups of miRNAs regulated by IAV infection could be defined: (1) miRNAs (*n* = 39) whose expression correlated with hemagglutinin (HA) mRNA expression and represented the general response to IAV infection independent of host genetic background; (2) miRNAs (*n* = 20) whose expression correlated with HA mRNA expression but differed between the two strains; and (3) remarkably, miR-147-3p, miR-208b-3p, miR-3096a-5p, miR-3069b-3p, and the miR-467 family, whose abundance even in uninfected lungs differentiated nearly perfectly (area under the ROC curve > 0.99) between the two strains throughout the time course, suggesting a particularly strong association with the differential susceptibility of the two mouse strains. Expression of subsets of miRNAs correlated significantly with peripheral blood granulocyte and monocyte numbers, particularly in DBA/2J mice; miR-223-3p, miR-142-3p, and miR-20b-5p correlated most positively with these cell types in both mouse strains. Higher abundance of antiapoptotic (e.g., miR-467 family) and lower abundance of proapoptotic miRNAs (e.g., miR-34 family) and those regulating the PI3K-Akt pathway (e.g., miR-31-5p) were associated with the more susceptible DBA/2J strain.

**Conclusion:**

Substantial differences in pulmonary miRNA expression between the two differentially susceptible mouse strains were evident even before infection, but evolved further throughout infection and could in part be attributed to differences in peripheral blood leukocyte populations. Thus, pulmonary miRNA expression both before and during IAV infection is in part determined genetically and contributes to susceptibility to IAV infection in this murine host, and likely in humans.

## Introduction

microRNAs (miRNAs) are short regulatory RNA molecules that occur in most eukaryotic cells and may have profound effects on a broad range of biological processes, including apoptosis, development, differentiation, proliferation, and immune responses (1–4). It, therefore, comes as no surprise that major changes in miRNA expression and activity have been shown to occur as part of the host cell response to infection by a variety of viruses (5–7). Increasing attention has been paid to the role(s) of miRNAs in influenza virus infection, with most studies having focused on influenza A virus (IAV). Studies have involved cells or tissues from natural hosts, such as humans (8–10), birds (11, 12), pigs (13), and macaques (14) and mice as adaptive hosts (15, 16). Major insights have been gained by comparing differences in the miRNome responses to IAV strains of differential pathogenicity (14–17) or by comparing effects of different host backgrounds (16). The importance of some of the differentially expressed miRNAs has also been validated experimentally [e.g., Buggele et al. (18)], thus underscoring that miRNAs may substantially influence the course of IAV infection (19). However, in spite of this ever growing interest in the interactions between IAV and the host miRNome, there have been no studies investigating differences in the miRNA response in hosts of well characterized differential genetic susceptibility to IAV. This is all the more surprising since recent work with inbred mouse strains of differential susceptibility to IAV infection clearly showed that the transcriptomic (mRNA) response to IAV is to a great extent determined genetically (20, 21). It is therefore quite plausible that major genetic differences might also manifest at the level of the miRNome’s response to IAV infection and that the description of this miRNome will enable identification of regulatory miRNA–mRNA networks that are critical for successful host defense against IAV infection and, perhaps, related viruses. Based on a well-established mouse model of IAV infection, and using deep sequencing of small RNAs (22), we have therefore used two inbred mouse strains of well characterized different genetic susceptibility to IAV infection to identify host strain-specific differences in the host miRNA response to this virus. We find substantial differences in the miRNA populations of the two strains, both at baseline and during infection, and an overall more rapid reprogramming in the more susceptible DBA/2J strain. The results suggest that host-strain-specific differences in abundance and regulation of miRNAs provide a link to host susceptibility to IAV infection.

## Materials and Methods

### Availability of Data

Small RNA sequencing data, a miRNA expression table, and tables with expression differences and correlations with hematological parameters in peripheral blood, including adjusted *p* values, are available in the Gene Expression Omnibus repository (http://www.ncbi.nlm.nih.gov/geo/query/acc.cgi?acc=GSE89064).

Data tables contain the 473 miRNAs that passed pre-analytic filtering. Columns within the table with expression differences (GSE89064_edgeR_miR.xls) represent the effect of infection or mock treatment compared to untreated mice, differences between infection and mock treatment at each time point, and differences between mouse strains after mock treatment, infection, or without treatment. All results were obtained with the edgeR package. Data table (GSE89064_miRNA_vs_blood_spearman_correlations.xlsx) contains Spearman correlation coefficients of all miRNAs with peripheral blood indices.

### Statement on Ethics Approval

All animal work was conducted according to the national guidelines of the animal welfare law of the Federal Republic of Germany and approved by the “Niedersächsisches Landesamt für Verbraucherschutz und Lebensmittelsicherheit, Oldenburg, Germany” (Permit Numbers: 3392 42502-04-13/1234 and 33.19-42502-04-13/1234).

### Virus

Original stocks of mouse adapted A/Puerto Rico/8/34 (H1N1, PR8) virus were obtained from Stefan Ludwig (University of Münster) (23). Virus stocks were propagated in the chorioallantoic cavity of 10-day-old pathogen-free embryonated chicken eggs for 48 h at 37°C as described previously (24).

### Animal Procedures

Infections were essentially carried out as described previously (20, 25). Briefly, female 12- to 13-week-old C57BL/6J and DBA/2J mice (*n* = 5–6 per time point and treatment) were anesthetized by intraperitoneal injection of 10 μl/g body weight of a stock solution of 0.5 ml ketamine (50 mg/ml, Invesa Arzneimittel GmbH, Freiburg, Germany), 0.5 ml 2% xylazine hydrochloride (Bayer Health-Care, Leverkusen, Germany), and 9 ml sterile NaCl 0.9% (Delta-Select GmbH, Dreieich, Germany). For intranasal infection, a viral dose of 2 × 10^3^ focus forming units (ffu) in a total volume of 20 μl sterile PBS was used. During the infection procedure, mice were held in the upright position and additional anesthetic was reinjected as needed. Mock treatment was identical to the real anesthesia/infections except that vehicle only (sterile PBS), not containing virus, was used for intranasal instillation. Mice were weighed on day 0 just before induction of anesthesia and on each subsequent day. Mice were killed by CO_2_ asphyxiation at 6, 12, 18, 24, 48, and 120 h with respect to infection or mock treatment. Untreated mice were used as *t* = 0 h control. Lungs were removed by cutting the five main bronchi and were washed in RNAlater RNA Stabilization Reagent (Qiagen Inc., Venlo, Netherlands), immediately after removal. After transfer into a new tube containing 2 ml RNAlater, lungs were stored overnight at 4°C and then at −20°C until further use.

### Hematological Measurements

An analysis of the hematological data from this time course experiment has been published separately (25). Briefly, 50 μl of whole blood were obtained by cardiac puncture and then analyzed with a VetScan HM5™ (Abaxis, Union City, CA, USA) hematology analyzer.

### RNA Isolation

Lungs were homogenized in 4 ml RLT buffer (Qiagen) containing 40 μl β-mercaptoethanol and stored in 450 μl aliquots at −80°C. After thawing, 450 μl of this suspension was mixed with 700 μl Qiazol (Qiagen), and all further steps of total RNA isolation were performed with the miRNeasy kit (Qiagen) according to the manufacturer’s recommendations.

### Small RNA Sequencing

Five samples per condition were submitted for small RNA sequencing. Sequencing was performed using the TruSeq™ Small RNA Sequencing Kit, Version of January 2011 (Illumina, Inc., San Diego, CA, USA). Approximately 1,250 ng of input RNA was used and the synthesized cDNA was amplified with 15 PCR cycles. cDNA library fragments with a length of about 150 nt were separated by gel electrophoresis and then isolated from the corresponding excised gel slice. Four to five biological replicates per condition were analyzed. Use of the Illumina molecular barcode allowed sequencing of eight samples per lane. To reduce batch effects, samples from the same time point and mouse strain were never run on the same lane. Sequencing was done using the HiSeq 2500 (Illumina Inc.) sequencer. The TruSeq™ Small RNA Sequencing Kit is specific for miRNAs and other small RNAs that have a 3′OH group resulting from enzymatic cleavage by RNA processing enzymes.

### Quantitative Real-time PCR (RT-qPCR)

Quantitative real-time PCR was performed with a LightCycler 480^®^ (La Roche AG, Basel, Switzerland) in 96-well plates in 20 μl reaction volumes, using 3 ng cDNA per reaction for miRNAs (miScript Reverse Transcription Kit, miScript SYBR Green PCR Kit; Qiagen) or 15 ng cDNA per reaction for detection of IAV hemagglutinin (HA) mRNA. Aliquots from the same RNA samples as for the sequencing experiment were used, with five biological replicates for each condition. HA expression was normalized against the mean of the Ct values of *Actb* and *Rpl4* mRNAs. Ct values of the miRNAs were not normalized against an internal control.

### Data Analysis

Illumina sequencing data were preprocessed using the CLC Genomics Workbench 6.04 (CLC Bio, Cambridge, MA, USA) and the resulting sequences were annotated using miRBase 19. Two additional downstream and upstream bases as well as two mismatches were allowed. 5′ and 3′ mature sequences (including variants) were used for further analysis. All subsequent analyses were done using the R environment and programming code (26). All *p* values were adjusted for false discovery rate (FDR).

Two samples were excluded before sequencing due to poor RNA quality/quantity, and three samples were excluded after sequencing due to low read numbers. Thus, small RNA sequencing data of 125 of the 130 originally submitted samples (96%) were available for analysis, amounting to five biological replicates for most conditions, but only four for the following conditions: untreated (0 h), mock treated (6 h), and infected (18 hpi) DBA/2J mice, and mock treated (6 and 120 h) C57BL/6J mice. A total of 1,276 miRNAs were detected. Only the 476 annotated sequences that were detected at a level of >1 count per million (CPM) in >5 samples were included in subsequent analyses. This did not change the number of total reads appreciably. Three miRNAs with strong outlier samples were removed because of an SD/median ratio >2.5 (calculated with cpm values of mouse strain and time post treatment). Line plots of these miRNAs did not show any relevant change in expression (data not shown).

Differentially expressed miRNAs were identified using generalized linear models contained in the edgeR package (27). For expression changes within a time course or differences between mock treated and infected mice, a fold change (FC) of 1.5 and *p* value of 0.05 were used as cutoff.

Differential miRNA expression in response to IAV infection was defined as follows: (1) significant (*p* ≤ 0.05) with respect to the untreated control (*t* = 0 h) and to the mock-treated mice at the same time point and (2) a ≥1.5 FC difference from the untreated mice. miRNAs that were differentially expressed due to the infection procedure, excluding the virus, were defined as follows: significant regulation after mock treatment and infection with respect to *t* = 0 h, but no significant expression difference between infected and mock samples from the same time point (*p* ≥ 0.05). A FC cutoff of ≥1.5 between untreated and mock-treated mice was used.

To compare absolute miRNA abundance in DBA/2J vs C57BL/6J mice, we used miRNAs that were differentially expressed in response to infection in at least one mouse strain and differed between the mouse strains, using the more rigorous FC cutoff of 2 and *p* ≤ 0.01. Furthermore, we defined a miRNA subset with no significantly different abundance between both mouse strains at all tested time points (ratio ≤ 1.5; *p* ≥ 0.1), but differential expression after IAV infection in either mouse strain. Clusters using k-means for longitudinal data were calculated using the kml package (28). Graphs were made using lattice (29), gplots (30), or ggplot2 (31).

### Functional Prediction

microRNAs that were found to differ between the two mouse strains were analyzed using three different approaches. From the significantly enriched pathways, we removed those that did not differ from putative pathways of miRNAs with similar abundance in both mouse strains. For the first approach, we used the DIANA mirPath v2 online tool (32) for annotated miRNA contained in miRBase 18. As settings, we used microT-CDS for target prediction with a threshold of 0.8. Results were visualized with a *p* value threshold of 0.05 using the option “pathways union.” This option shows the contribution of each miRNA to the pathways enrichment, showing only miRNAs whose targets are significantly (*p* ≤ 0.05) enriched for that pathway. From the significant pathways, we removed those with a 1.5-fold lower number of significant miRNAs for the same pathway in the control group of miRNAs with similar abundance between the two mouse strains.

For the second strategy, we used putative miRNA targets that were found in at least three of four databases (MicroCosm, PITA, miRanda and miRDB) [MicroCosm (33); miRanda (34); miRDB (35); PITA (36)]. Targets that were shared by at least two miRNAs were chosen for functional analysis.

Using the R packages “goseq” and “GO.db,” we identified significantly enriched GO Terms using putative targets of all 473 detected miRNAs as background. Of the significantly enriched terms for host strain-dependent miRNAs, we chose only those which had at least 1.5-fold more targets for a pathway, compared to the control group with similar miRNA abundance, or that were not found in the control group.

Putative miRNA targets of step 2 were analyzed using the online tool DAVID (Database for Annotation, Visualization and Integrated Discovery) Bioinformatics Resources 6.7 (37, 38) and the *Mus musculus* genome as background. For functional observations, we used default settings. Results of the DAVID tool included a Functional Annotation Table with functional terms for each gene; a Functional Annotation Chart that calculates the enrichment of each term and a Functional Annotation Clustering that clusters Terms that are related. Of the Functional Annotation Table, we added the options fold enrichment and FDR. Functional Annotation Charts were downloaded and loaded into the R program to allow direct comparisons of different enrichment tables. To identify functions of miRNAs with different abundance in DBA/2J and C57BL/6J mice, only those significantly enriched terms (FDR ≤ 0.05) were chosen that were found for miRNAs with different, but not for miRNAs with similar abundance in both mouse strains or were at least 1.5-fold more highly enriched.

### Other Statistical Analyses

For pairwise comparisons of miRNA or mRNA Ct values, we used Tukey’s Honest Significant Differences Test for homogeneous variances. We used a significance threshold of *p* ≤ 0.05. Receiver operating characteristic (ROC) curve analysis was performed using the R package ROCR (39) and counts per million (cpm) data.

## Results

### Brisker Progression of Infection and More Pronounced Weight Loss in the DBA/2J Mouse Strain

**Figure 1:**
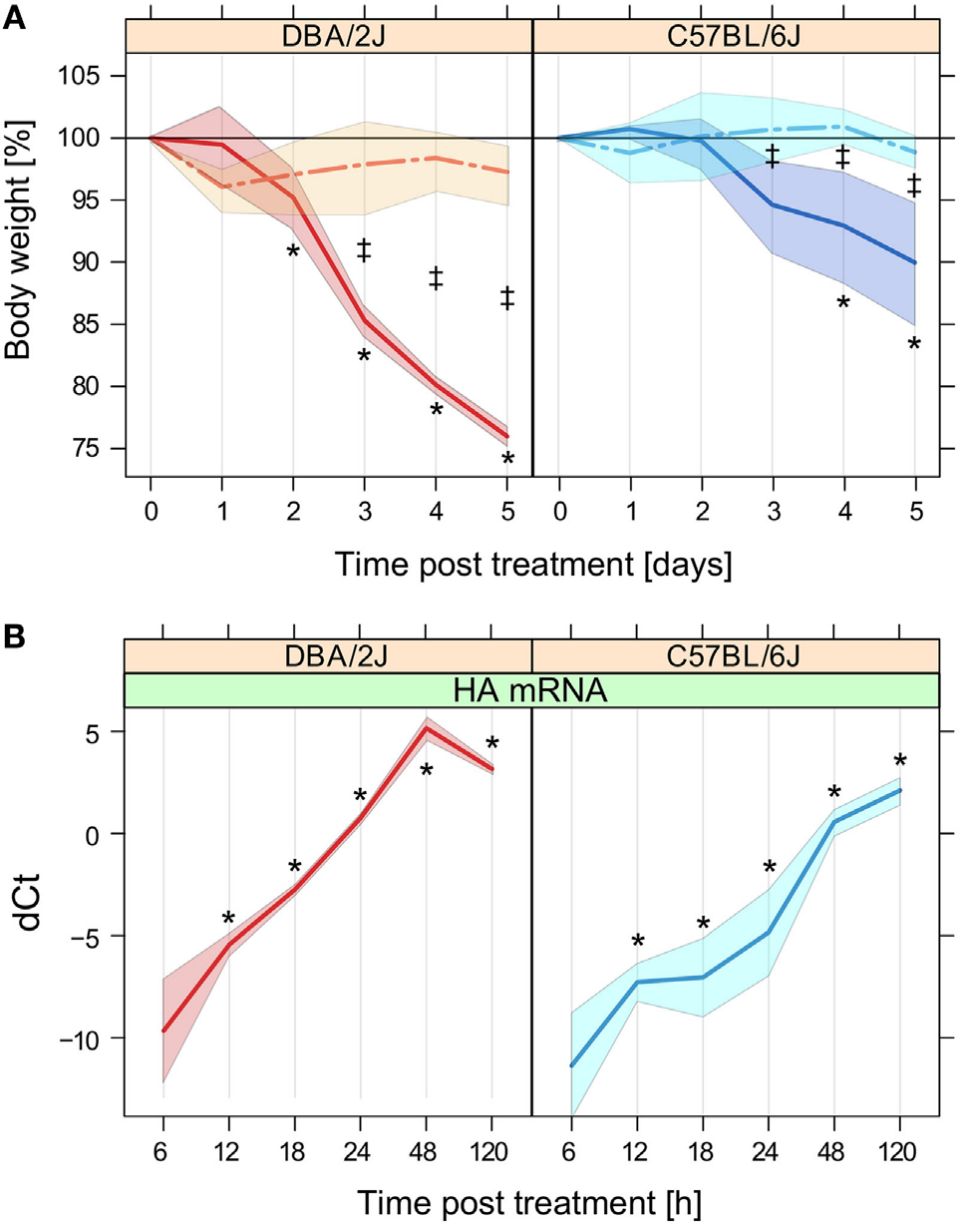
**Weight loss and expression of influenza A virus (IAV) hemagglutinin (HA) mRNA**. Weight loss and HA mRNA expression throughout the 5-day time course after mock treatment or infection with IAV strain PR8 as outlined in Section “Materials and Methods.” **(A)** Weight loss, expressed as the mean percentage of body weight measured at *t* = 0 h before administration of anesthesia. No mice had to be killed because of >30% weight loss. Data are based on the mice that were sacrificed at the 120 h time point (*n* = 5–6 per group). **(B)** Relative quantification of IAV HA mRNA in mouse lung by RT-qPCR in the 5-day time course shown in panel A. dCt refers to Ct_reference_ − Ct_target mRNA_, where Ct_reference_ is the arithmetic mean of the Ct values of β-actin and Rpl4. As expected, HA mRNA was not detected in the mock-treated mice (data not shown). Data are based on five mice per group except for DBA/2J at 18 h (*n* = 4). Solid lines, infection; interrupted lines, mock treatment. Left panels, DBA/2J strain; right panels, C57BL/6J strain. The colored areas around the lines indicate SD. Note that the *x*-axes of the panels are based on different scales. *p ≤ 0.05 for difference with respect to *t* = 0 h; ^‡^*p* ≤ 0.05 for difference between mock-treated and infected mice at the given time point (Tukey’s test). This figure is adapted from two articles published by BioMed Central (21, 25), which describe other aspects of this time course experiment.

Higher weight loss, compared to less susceptible mouse strains, is a hallmark of the high susceptibility of DBA/2J mice to IAV infection. Consistent with our previous observations (40), marked weight loss was observed after infection of this strain, which reached a maximum of approximately 25% by day 5 (Figure 1A). As shown previously (40), weight loss of the less susceptible strain, C57BL/6J, was less pronounced, as reflected in a mere 5–10% reduction by day 4–5. There was no significant weight loss among the mock-treated mice between day 1 and 5.

Quantitative real-time PCR revealed a brisk rise of mRNA encoding IAV HA in lungs of both mouse strains after infection (Figure 1B). HA mRNA was detected at low levels as early as 6 hpi in both strains, followed by a rapid rise that peaked at 48 and 120 hpi in DBA/2J and C57BL/6J mice, respectively. HA mRNA levels were significantly higher in DBA/2J than in C57BL/6J. This difference was significant as early as 12 hpi with a ratio DBA/2J to C57BL/6J of 3.6:1 and increased to a constant ratio of about 23 to 24:1 between 18 and 48 hpi, which diminished markedly by 120 hpi due to a decline in HA transcript levels in the DBA/2J strain by this time point. Thus, the infection evolved more briskly and peaked sooner in the more susceptible DBA/2J strain, with differences manifesting as early as 12 hpi.

### Global Changes in miRNA Expression throughout the Time Course

**Figure 2:**
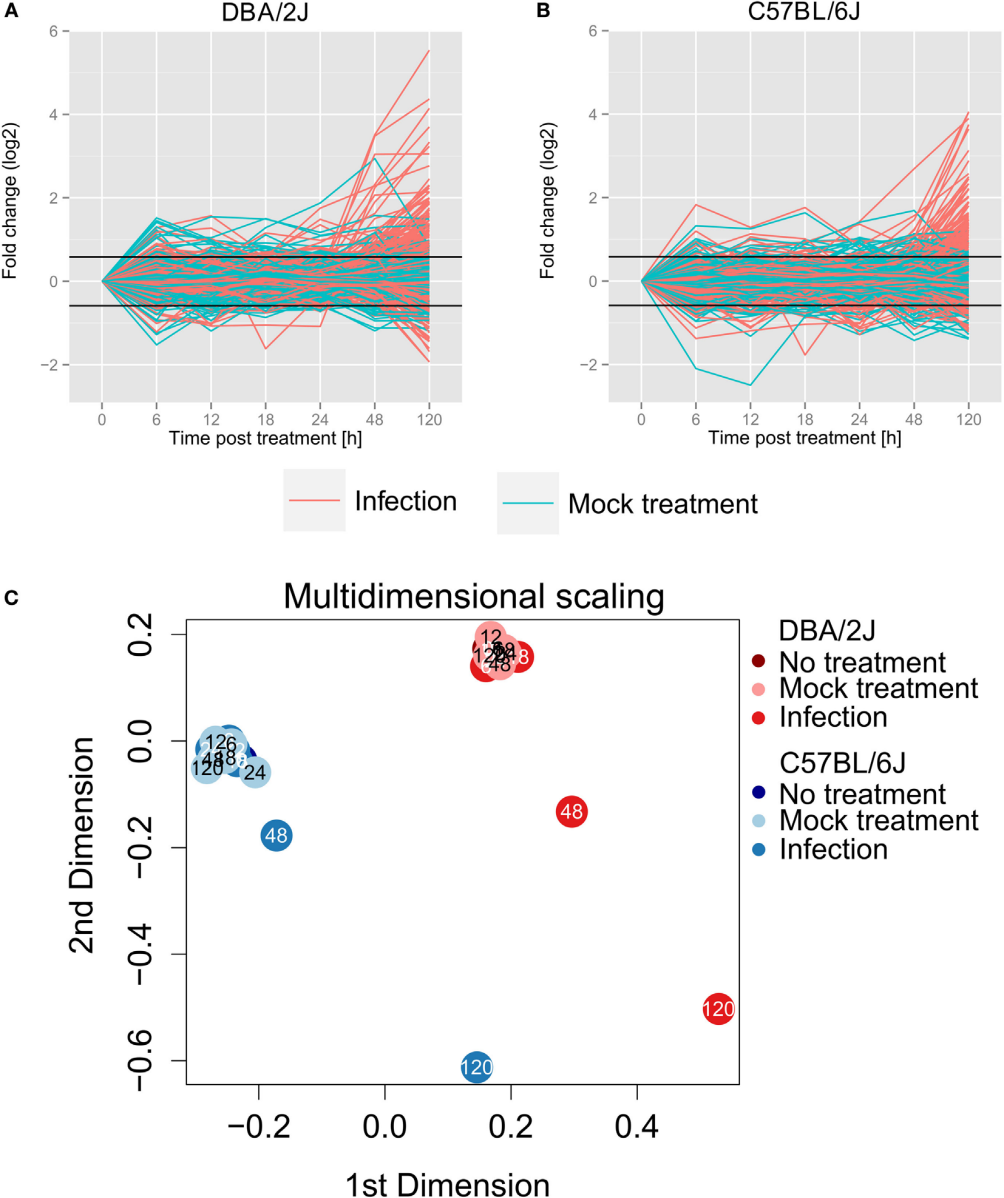
**Global miRNA expression changes throughout the 120 h time course of influenza A virus infection**. Values of all 473 miRNAs that passed preanalytical filtering are shown in panels **(A,B)** (turquoise lines, mock treatment; red lines, infection). **(A,B)** change in expression (log2); FC was calculated with the R package edgeR (27). **(A)** DBA/2J strain; **(B)** C57BL/6J strain. **(C)** Multidimensional scaling based on the expression data of all 473 detected miRNAs (edgeR package). The data, as well as the data shown in Figures 3–6, are based on five mice per condition, except that four mice were available for untreated (0 h), mock treated (6 h), and infected (18 h) DBA/2J mice, and for mock treated (6 and 120 h) C57BL/6J mice.

Eighty-eight percent (10.7 million on average) of all obtained sequences corresponded to annotated miRNAs. A total of 473 miRNA species passed pre-analytic data processing and were selected for subsequent analyses. In both mouse strains, analysis of the 473 selected miRNAs (Figures 2A,B) revealed a narrow range in miRNA expression FC during the time course. Accordingly, multidimensional scaling (MDS, Figure 2C) revealed that samples from mock-treated and infected mice at any time point between 6 and 24 h formed two tight clusters (one for each mouse strain) with the respective uninfected (*t* = 0 h) mice. In the DBA/2J mice, an apparent weak up and downregulation of some miRNAs due to the mock treatment was seen at 6 h (Figures 2A,B), but this was not statistically significant, and there was no apparent tendency toward a mock effect in the C57BL/6J strain. Thus, the infection/anesthesia procedure did not induce significant changes in miRNA populations in either mouse strain. Of note, a marked difference in miRNomes between the strains was apparent even in the uninfected lungs (Figure 2C). These strain-specific differences dominated the global differences in miRNomes throughout the time course, which remained relatively constant.

In both mouse lines, line plots (Figures 2A,B), MDS (Figure 2C), and Euclidian distance dissimilarity analysis (EDDA, data not shown) consistently indicated a global miRNA reprogramming beginning at 48 hpi, followed by a pronounced further increase at 120 hpi. The overall mean FC was constant within the first 24 h and increased in DBA/2J and C57BL/6J mice after 48 h and 120 h, respectively. As indicated by MDS and confirmed using EDDA, miRNome reprogramming at 48 hpi was greater in the DBA/2J than the C57BL/6J strain, whereas the two strains reached a similar overall degree of reprogramming by 120 hpi. Notably, HA mRNA expression in DBA/2J mice decreased between 48 and 120 hpi (Figure 1B), but miRNome reprogramming increased further during the same time frame. Therefore, host miRNA reprogramming did not seem to be coupled directly to viral HA RNA expression.

**Figure 3:**
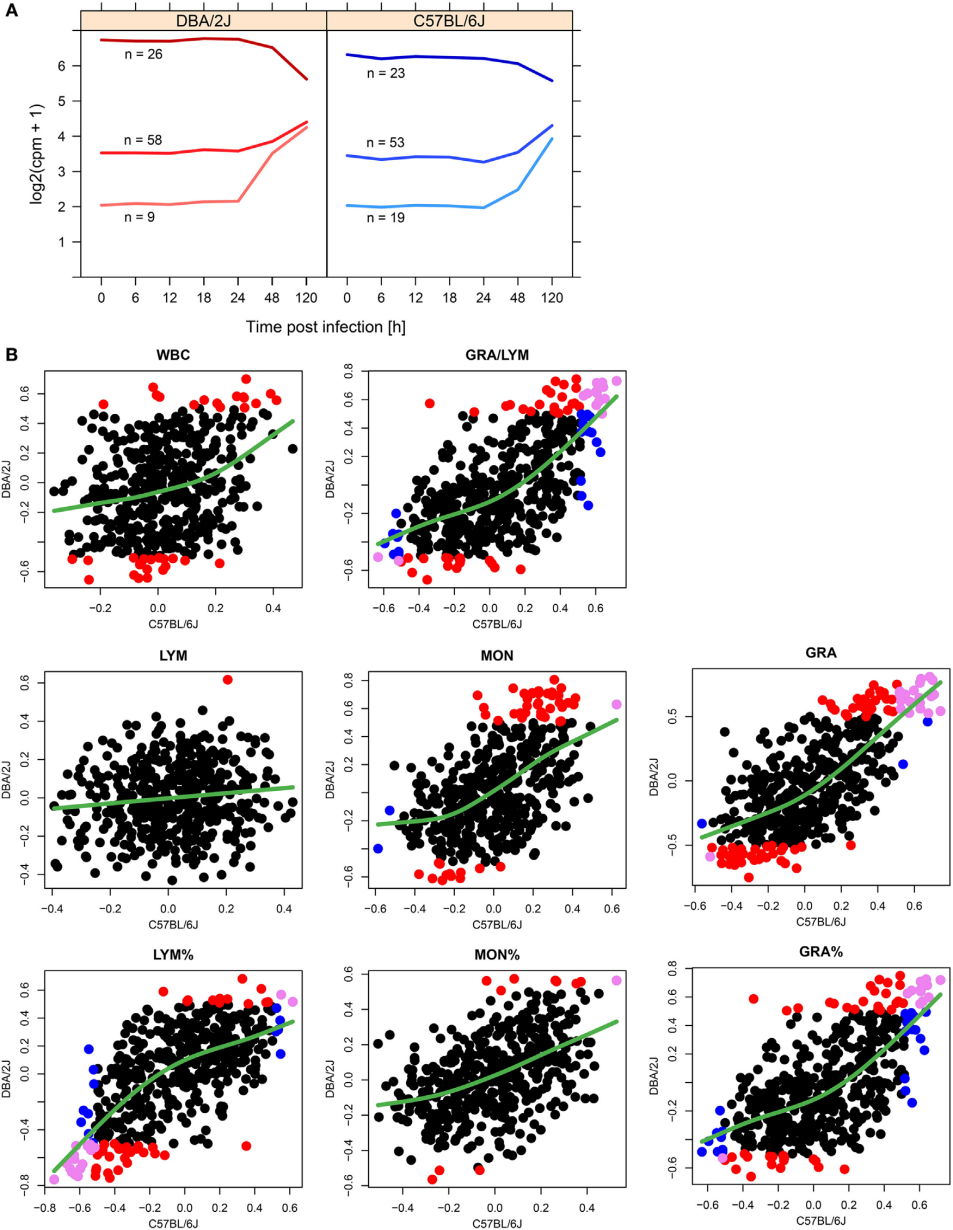
**Potential contributions of infiltrating leukocytes to pulmonary miRNome reprogramming**. **(A)** Line plots visualizing the associations between miRNA abundance and direction of regulation of miRNAs regulated in response to influenza A virus (IAV) infection (115 different miRNA species, FC ≥|1.5|, FDR adjusted *p* value ≤ 0.05 in either mouse strain, see Figure 4). The clusters were identified with the k means of longitudinal data (kml) package of R (28) using the FC (log2) data of IAV infected mice compared to untreated (*t* = 0 h) mice. **(B)** Correlation of miRNA expression with peripheral blood leukocyte parameters. Using data from infected and control (uninfected) mice, expression (cpm) of 473 miRNAs was correlated with hematological parameters of the same mice. Significant correlations (Spearman correlation; ρ ≥|0.5| and *p* ≤ 0.05) are indicated in different colors according to mouse strain: red, significant correlation in DBA/2J only; blue, significant correlation in C57BL/6J only; violet, significant correlation in both strains. The green smoothing line was generated using the R function smooth.spline (stats package) using default settings. WBC, white blood cells; LYM, LYM%, absolute and relative lymphocyte counts; MON, MON%, absolute and relative monocyte counts; GRA, GRA%, absolute and relative granulocyte counts.

To test whether there was any association between the initial expression level of the infection-regulated miRNAs and the direction of their regulation, the 115 infection-regulated miRNAs (differentially expressed in DBA/2J or C57BL/6J mice with respect to mock-infected mice; FC ≥|1.5|, FDR adjusted *p* value ≤ 0.05) were grouped into three clusters according to baseline expression level and direction of regulation (Figure 3A). Indeed, a negative correlation between initial expression and direction of regulation was identified in both mouse strains: downregulated miRNAs tended to have a high baseline expression level, intermediately upregulated miRNAs an intermediate baseline expression level, and strongly upregulated miRNAs the lowest. The three clusters converged toward 120 hpi, at a point corresponding to approximately five normalized and log2 transformed reads (log2 counts per million; cpm). Thus, the direction of regulation of the miRNAs depended on their initial abundance (expression level). These observations are consistent with a model in which miRNAs with high baseline expression are predominantly expressed in resident lung cells, but miRNAs of low initial abundance are mainly expressed in immune cells and their abundance increases during infection due to their immigration into the lung combined with the corresponding changes in their activation state.

To assess the contributions of changes in immune cell populations to changes in miRNA expression, we quantified correlations between peripheral blood leukocyte parameters and expression of each of the miRNAs (Figure 3B). Overall, higher numbers of significant correlations were measured in DBA/2J mice [compare red (DBA/2J) and and blue (C57BL/6J) circles in Figure 3], which was particularly pronounced in total leukocyte (WBC) and monocyte counts. Absolute granulocyte numbers and miRNA expression correlated most significantly in both mouse strains (23 miRNAs with significant correlations; violet circles in Figure 3), with miR-223-3p, miR-142-3p, and miR-20b-5p correlating the most positively in both mouse strains ([ρ_DBA/2J_ + ρ_C57BL/6J_]/2 > 0.7). There was only a negligible correlation (one miRNA in DBA/2J mice) with absolute lymphocyte counts. To test the above stated hypothesis that pulmonary miRNAs expressed in infiltrating immune cells are initially expressed at low level in the lung, we asked whether there was an association between miRNA correlation with peripheral blood indices and initial miRNA abundance. Indeed, miRNAs that correlate positively (ρ ≥ 0.2) with MON, GRA, GRA/LYM, and GRA% had a lower initial abundance (≥1.5-fold lower; *p* value ≤ 0.01, Wilcoxon test) than miRNAs that correlate negatively (ρ ≤ −0.2). Taken together, these results suggest that expression of pulmonary miRNAs during the first 120 h of infection may be in part explained by infiltration with myeloid immune cells, particularly in the more susceptible DBA/2J strain.

### Mouse Strain-Specific Differences in miRNome Regulation after IAV Infection

**Figure 4:**
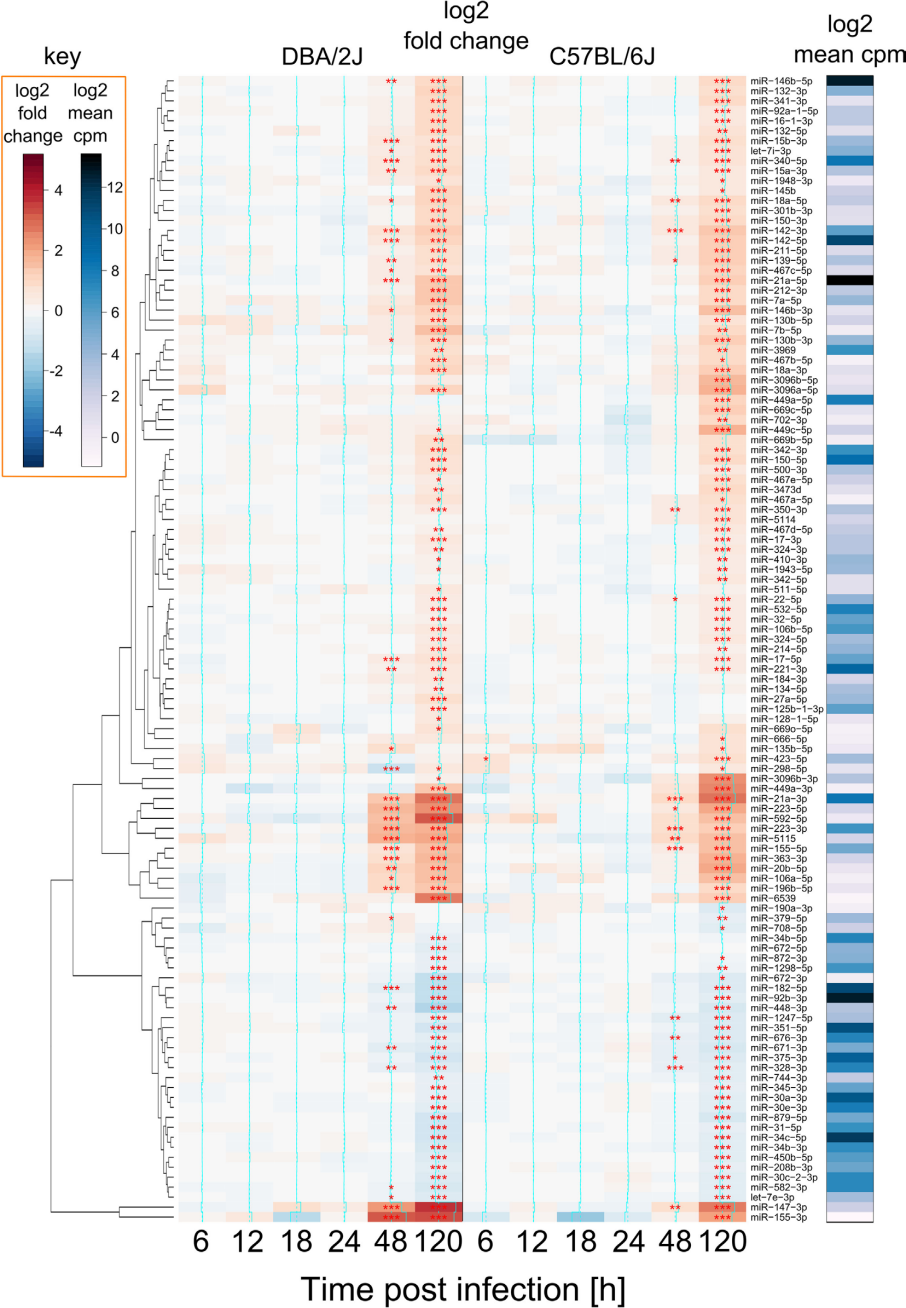
**miRNAs regulated during influenza A virus (IAV) infection**. Heat map showing all 115 miRNAs that were differentially expressed after IAV infection (see Materials and Methods) of DBA/2J and/or C57BL/6J mice. FC data and *p* values (FDR adjusted) represent the difference between infected and mock-treated mice. Red: upregulated; blue: downregulated. The right bar indicates log2 counts per million (edgeR package) of these miRNAs in DBA/2J and C57BL/6J mice together, ranging from low (white) and mid (blue) to high (black) abundance. Expression of 93 miRNAs changed during the infection in the DBA/2J strain and 95 in the C57BL/6J strain, corresponding to a total of 115 regulated miRNAs. Seventy-three of these were regulated in both strains (***p value ≤ 0.001; **p value ≤ 0.01; *p value ≤ 0.05). Stars are only shown for differences between infected and mock-treated mice and were also included for significant miRNAs with an FC <|1.5|.

As mentioned above, 115 miRNAs were significantly regulated after infection. The majority (75%) were upregulated and only about 25% were downregulated. Of note, the number of differentially expressed miRNAs and their FC direction was similar in both mouse strains (Figure 4), i.e., miRNAs that were upregulated in one strain, were also upregulated in the other, and *vice versa*. In both strains, a first significant response to the infection was apparent at 48 hpi, with a slightly higher number of differentially expressed miRNAs and higher FC values being observed in the DBA/2J strain than in the C57BL/6J strain. An at least twofold greater induction in C57BL6/J mice was detected in eight miRNAs (miR-190a-3p, miR-449a-5p, miR-449a-3p, miR-449c-5p, miR-3096a-5p, miR-3096b-5p, miR-3096b-3p, and miR-669c-5p). Consistent with MDS and EDDA, the two strains reached a similar overall degree of reprogramming by 120 hpi, but differences at the earlier time points were greatest at 48 h.

**Figure 5:**
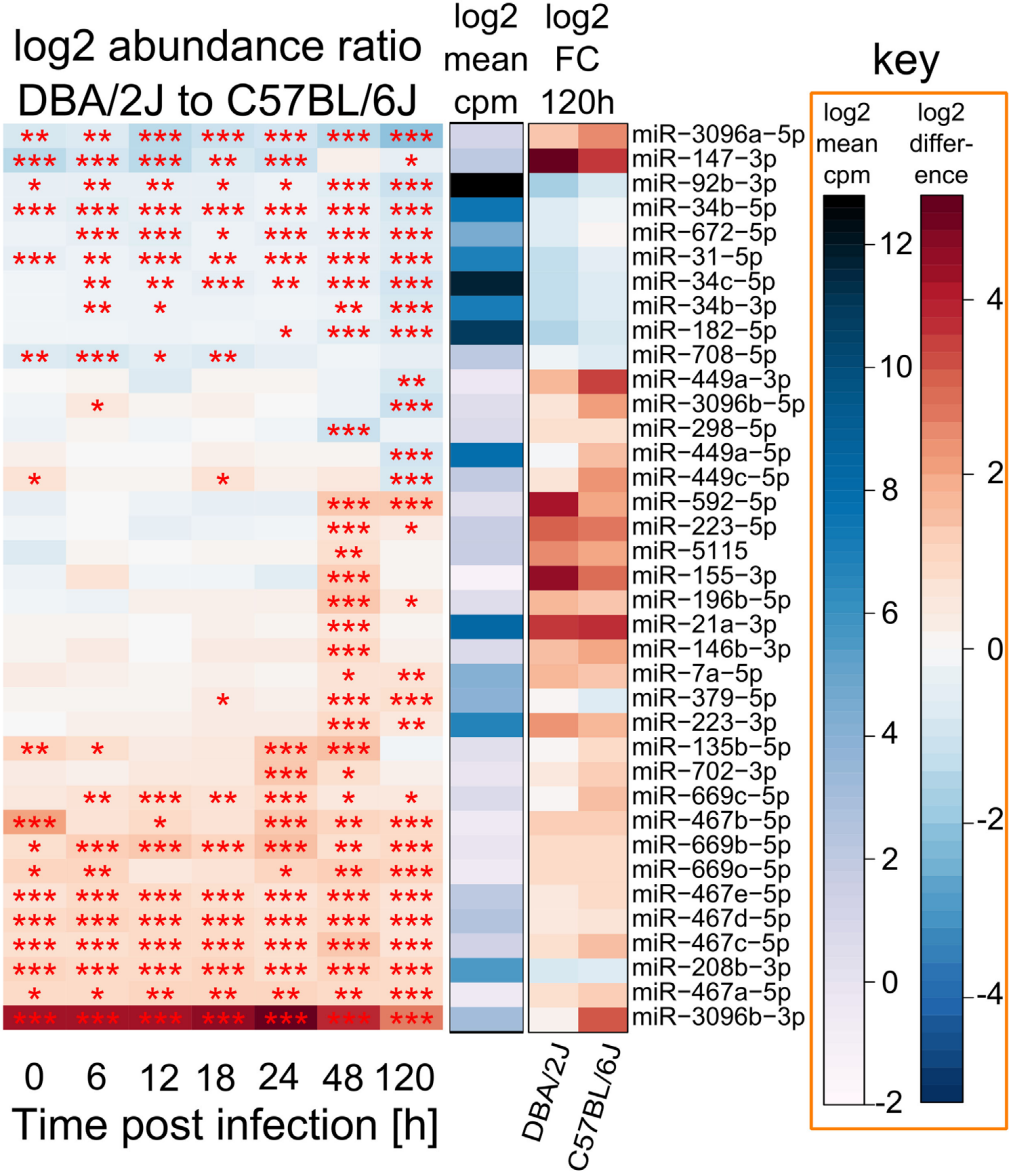
**miRNAs that differ in absolute abundance between DBA/2J and C57BL/6J mice**. Depiction of 37 miRNAs with a differential abundance of ≥|2| and a *p* value (FDR adjusted) of at ≤0.01 between DBA/2J and C57BL/6J mice throughout the time course. Red colors in the heat map indicate miRNAs that are more abundant in DBA/2J mice (22 miRNAs), blue colors indicate miRNAs that are more abundant in C57BL/6J mice (15 miRNAs). The 115 miRNAs that are infection dependently regulated in DBA/2J or C57BL/6J mice (Figure 4) were used as input miRNAs. The blue bar indicates mean miRNA abundance across all samples (log2 cpm). The FC of these miRNAs at 120 hpi is shown using shades of blue (low DBA/2J vs C57BL/6J ratio, i.e., higher abundance in C57BL/6J mice) and shades of red (high DBA/2J vs C57BL/6J ratio, i.e., higher abundance in DBA/2J mice) (***p value ≤ 0.001; **p value ≤ 0.01; *p value ≤ 0.05). Stars were also included for significant miRNAs with an absolute ratio <2.

In a next step, we tried to identify miRNAs that might contribute to the differences in miRNomes between the two mouse strains revealed in Figure 2 and, thus, might affect host strain susceptibility. Abundances of a subset of 37 infection-regulated miRNAs differed (FC ≥|2|, FDR adjusted *p* value ≤ 0.01) between the two mouse strains at any of the tested time-points (Figure 5), 22 of which were more abundant in DBA/2J and 15 more abundant in C57BL/6J mice. Six of these (16%) were significantly infection-regulated in DBA/2J only and 10 (27%) in C57BL/6J only. Pulmonary expression of eight miRNAs differed between the two mouse strains even before infection. Expression of 10 miRNAs differed significantly between the two strains between 6 and 18 hpi, expression of 13 miRNAs at 24 hpi, expression of 20 miRNAs at 48 hpi, and expression of 23 miRNAs at 120 hpi. The miRNAs whose expression was lower/higher in DBA/2J mice in the uninfected state mainly continued to be expressed at a lower/higher level after infection, even after 5 days. Thus, two major patterns could be discerned among the host strain-dependently regulated miRNAs: (1) miRNAs that were differentially expressed in the two strains throughout the entire time course, and (2) miRNAs whose differential expression manifested at the later time points. Most miRNAs of group two were more abundant in DBA/2J mice. miR-147-3p constituted an exception in that it was markedly overexpressed in C57BL/6J mice within the first 24 hpi and had a similar expression level as DBA/2J at 48 hpi. miR-3096b-3p and miR-3096b-5p showed the strongest host strain-dependent difference in both directions. The DNA Intelligent Analysis (DIANA), the Gene Ontology analyzer for RNA-seq data (R package goseq), and the Database for Annotation, Visualization, and Integrated Discovery (DAVID) were then used to search for putative mRNA targets of miRNAs whose expression was different between the two mouse strains (Figure 5) and the functional pathways putatively associated with these mRNAs. Among the pathways relating to immune functions, *Fc gamma R-mediated phagocytosis, HIF-1 signaling pathway, positive regulation of protein kinase B signaling cascade, SH3 binding*, and *leukocyte differentiation* were significantly over-represented.

There were also distinctive features within each of the two major miRNA expression patterns mentioned above. For instance, miRNAs whose abundance did not differ between uninfected and incipiently infected mice tended to be more highly expressed in DBA/2J mice at 48 hpi. Furthermore, seven miRNAs were expressed more highly in C57BL/6J mice and were mainly downregulated across the time course (miR-92b-3p, miR-34b-5p, miR-672-5p, miR-31-5p, miR-34c-5p, miR-34b-3p, and miR-182-5p; listed in descending order according to the heat map in Figure 5). Although these miRNAs were less abundant in DBA/2J mice, they were downregulated even more strongly in this strain. In addition, the absolute abundance of these miRNAs was relatively high, suggesting that they are expressed in resident lung cells and not in infiltrating immune cells. Indeed, in DBA/2J all seven miRNAs (and four of seven in C57BL/6J) belong to the upper trajectory cluster in Figure 3. By using functional enrichment of putative targets, *PI3K-Akt signaling pathway, Notch signaling pathway, hemopoiesis, leukocyte differentiation, natural killer cell mediated cytotoxicity*, and different cancers were among the enriched functions. When considering all 37 infection-regulated miRNAs whose expression differed between the two mouse strains, nine miRNAs had targets that were significantly over-represented in the *PI3K-Akt signaling* pathway (DIANA tool). Five of these [miR-34b-3p, miR-34c-5p, miR-34b-5p, miR-92b-3p, and miR-182-5p; as well as miR-31-5p, which was identified through literature search (41)] belonged to the aforementioned seven miRNAs which were expressed more highly in the C57BL/6J mice and downregulated throughout the time course.

### miRNAs That Are More Abundant in the C57BL/6J Strain Contain More Putative Binding Sites for IAV RNA

To test whether these 37 miRNAs (Figure 5) might contribute to the higher IAV resistance of C57BL/6J mice, we searched for putative binding sites for them in the IAV genome. Using the ViTa Database, the human homologs of miR-135b-5p, miR-147-3p, miR-31-5p, miR-379-5p, miR-7a-5p, as well as the miR-449 (-5p) and miR-34 (-5p) families, were predicted to bind to viral RNA segments of influenza A/Puerto Rico/8/34/Mount Sinai (H1N1). Most of these miRNAs were more abundant in C57BL/6J mice (*p* = 0.013, hypergeometric test) and might, therefore, contribute to the higher resistance of C57BL/6J mice to IAV infection.

### Potential Involvement of Apoptotic Processes in Host-Strain Susceptibility to IAV Infections

**Figure 6:**
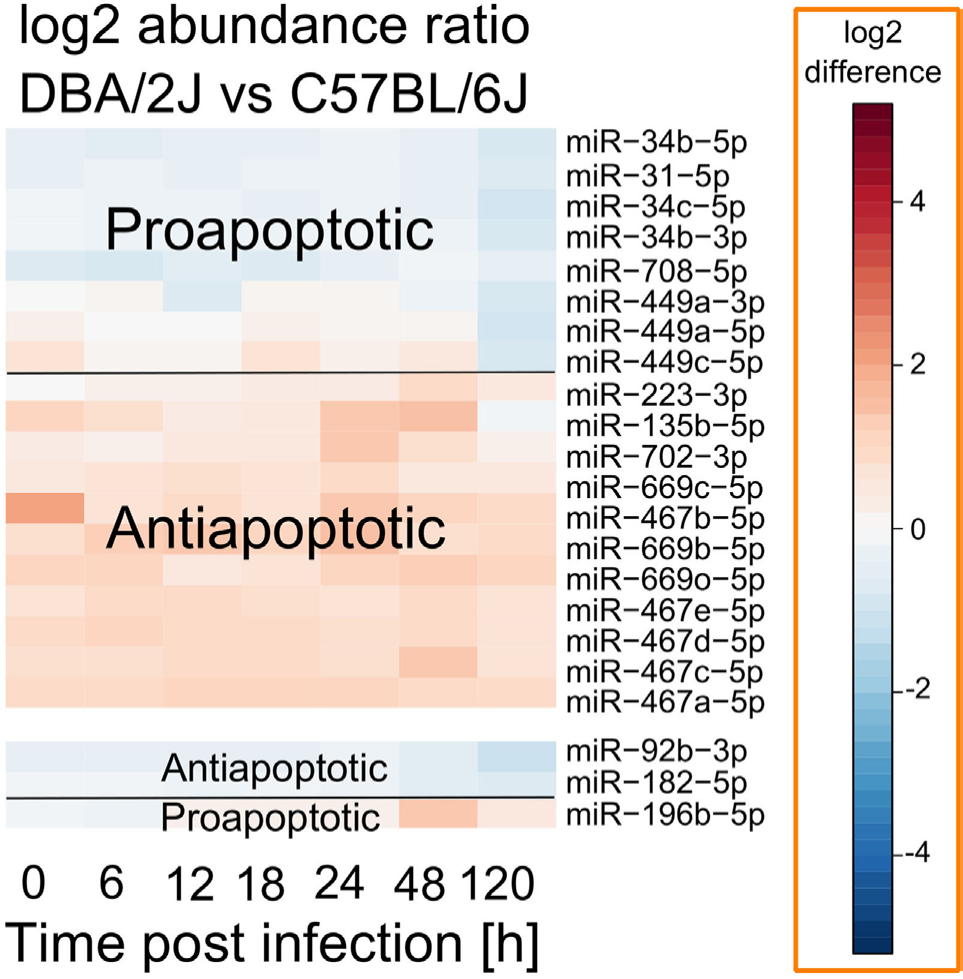
**Apoptosis-related miRNAs that differ in absolute abundance between DBA/2J and C57BL/6J mice**. Data are based on those shown in Figure 5. Red, higher expression in DBA/2J mice; blue, higher expression in C57BL/6J mice. Functional information was found by literature research as described in the text above.

Many miRNAs whose expression differed between DBA/2J and C57BL/6J mice during infection belong to the miR-467, miR-449, and miR-34 families. Of note, all three families have been shown to play roles in apoptosis (42–44), and several publications have demonstrated the relevance of apoptosis to IAV infection (17, 45–47). We therefore tested whether there might be an association between strain-specific miRNA expression and apoptosis. As shown in Figure 6, miRNAs that were more highly expressed in the susceptible DBA/2J strain tended to be antiapoptotic, whereas miRNAs that were more highly expressed in the resistant C57BL/6J strain tended to be pro-apoptotic (*p* = 0.002, two-tailed Fisher exact test) (42–44, 48–53).

### IAV-Regulated miRNAs with Host-Strain-Specific Expression in the Absence of Infection

**Figure 7:**
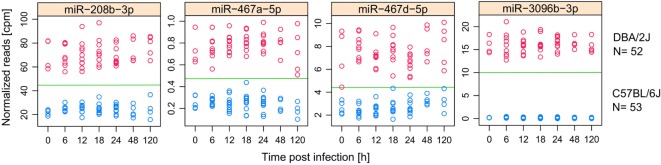
**miRNAs with consistently different expression in DBA/2J and C57BL/6J mice**. miRNAs that have an AUC = 1 for the separation between uninfected, mock treated, and incipiently infected (6–24 h) DBA/2J from C57BL/6J mice. Data are based on 52 DBA/2J mice (4 at 0 h, 10 at 12 h, 9 at 18 h, 10 at 24 h, and 5 at 48 and 120 h) and 53 C57BL/2J mice (5 at 0 h, 10 at 12, 18 and 24 h, 5 at 48 h, and 4 at 120 h). Dots indicate the normalized (cpm) miRNA expression in DBA/2J (red) and C57BL/6J (blue) mice. All shown miRNAs are differentially expressed in at least one mouse strain after influenza A virus infection (Figure 4).

Considering the differences between the pulmonary miRNomes of the two mouse strains even before IAV infection (Figure 2C), we used receiver operating characteristic (ROC) curve analysis to identify miRNAs whose expression levels differed consistently between the two mouse strains in the absence of IAV infection and which might, therefore, be used in the uninfected state to predict intrinsic host susceptibility. To increase the sample size and, therefore, statistical power, this analysis comprised all treatments and time points that did not show evidence of infection-dependent miRNA expression changes, i.e., untreated mice (0 h), mock-treated mice (6–120 h), and infected mice in the first 24 hpi (6–24 h). This amounted to a sample size of 52 DBA/2J mice and 53 C57BL/6J mice. Five miRNAs were identified with areas under the ROC curve (AUC) = 1, indicating that there was no overlap in their expression levels between the two strains, and expression of four of them changed during infection (Figure 7). In total, there were eight differentially expressed miRNAs with AUCs > 0.99 (miR-147-3p, miR-208b-3p, miR-467a-5p, miR-467c-5p, miR-467d-5p, miR-467e-5p, miR-3096a-5p, and miR-3096b-3p) whose abundance changed significantly during infection. Most of these highly strain-specifically expressed miRNAs (except miR-147-3p and miR-3069a-5p) were expressed more highly in the DBA/2J strain. Overall, miRNAs belonging to the miR-467 family were over-represented in this group of strain-specific miRNAs: 11 miRNAs of this family were among the 473 miRNAs that passed initial data filtering (see Materials and Methods), and nine of them had AUCs > 0.946. Taken together, the above results suggest (1) that the selective expression of several IAV-regulated miRNAs, including miR-147-3p, miR-208b-3p, miR-3096a-5p, miR-3069b-3p, and the miR-467 family, in uninfected and incipiently infected DBA/2J lungs was specifically associated with higher susceptibility of this mouse strain to IAV infection at later time points and (2) that it accurately predicted this higher susceptibility.

### Technical Validation of RNAseq Results with RT-qPCR

**Figure 8:**
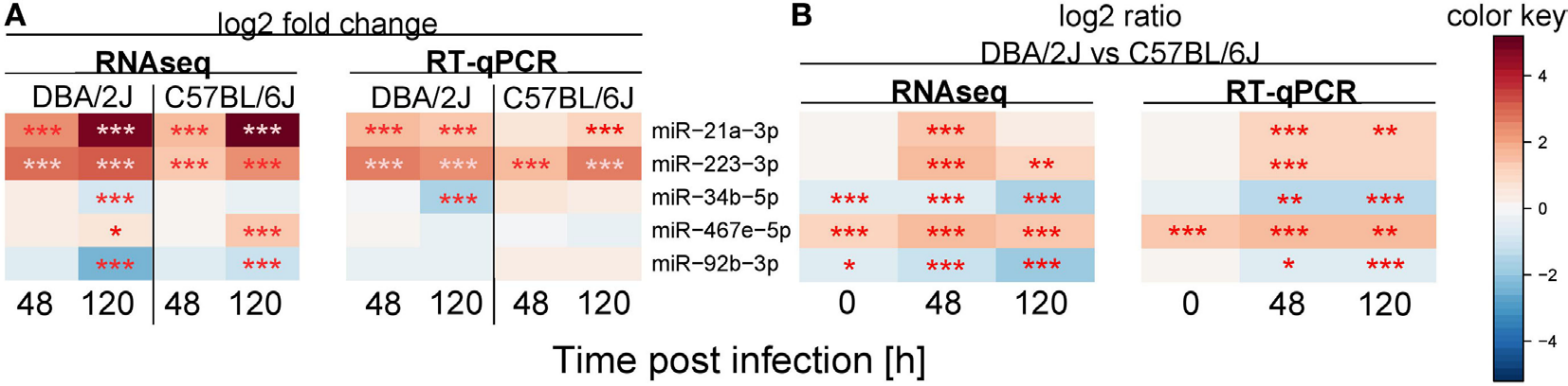
**Validation of miRNA expression using quantitative real-time PCR (RT-qPCR)**. RT-qPCR validation experiment for five selected miRNAs (compare Figures 4 and 5). The same mouse lungs were used as for the sequencing experiment. Colors indicate **(A)** mean log2 fold change expression with respect to the untreated control or **(B)** miRNA expression differences between the DBA/2J and the C57BL/6J mouse strain. Red: **(A)** upregulated miRNA, **(B)** higher expression in DBA/2J mice; blue: **(A)** downregulated miRNA, **(B)** higher expression in C57BL/6J mice (***p value ≤ 0.001; **p value ≤ 0.01; *p value ≤ 0.05). All *p* values were false discovery rate adjusted.

Five miRNAs were chosen that exemplify three patterns of expression differences between DBA/2J and C57BL/6J mice. These are comprised of miRNAs with a lower (miR-34b-5p and miR-92b-3p) or higher (miR-467e-5p) expression in DBA/2J vs C57BL/6J mice throughout the time course and those that are more highly expressed at 48 or 120 hpi only (miR-223-3p and miR-21a-3p). RNA from the same mice was used for both analyses. When expressing the RNAseq data as CPM, RT-qPCR data of four of the five miRNAs correlated strongly with the RNAseq data (ρ = 0.8 each, *p* ≤ 0.05, Spearman correlation), whereas miR-92b-3p did not correlate significantly (ρ = 0.25, *p* = ≥ 0.05). When FC values were used, RT-qPCR detected changes in miR-21a-3p, miR-223-3p, and miR-34b-5p expression in the same direction as measured by RNAseq, whereas no significant regulation was observed for miR-467e-5p and miR-92b-3p (Figure 8A). The RT-qPCR analysis confirmed the higher expression of miR-223-3p, miR-21a-3p, and miR-467e-5p in DBA/2J and the lower expression of miR-34b-5p and miR-92b-3p after infection, compared to C57BL/6J (Figure 8B). The RT-qPCR data differed in a minor way from the RNAseq data in that expression of miR-34b-5p and miR-92b-3p at *t* = 0 did not differ significantly between the mouse strains (Figure 8B). Taken together, these results suggest that there is substantial, but not perfect, agreement between the RT-qPCR and RNAseq data, thus validating our approach to use RNAseq for miRNA profiling.

### Identification of miRNA Subpopulations Whose Expression Correlates with HA mRNA Expression

**Figure 9:**
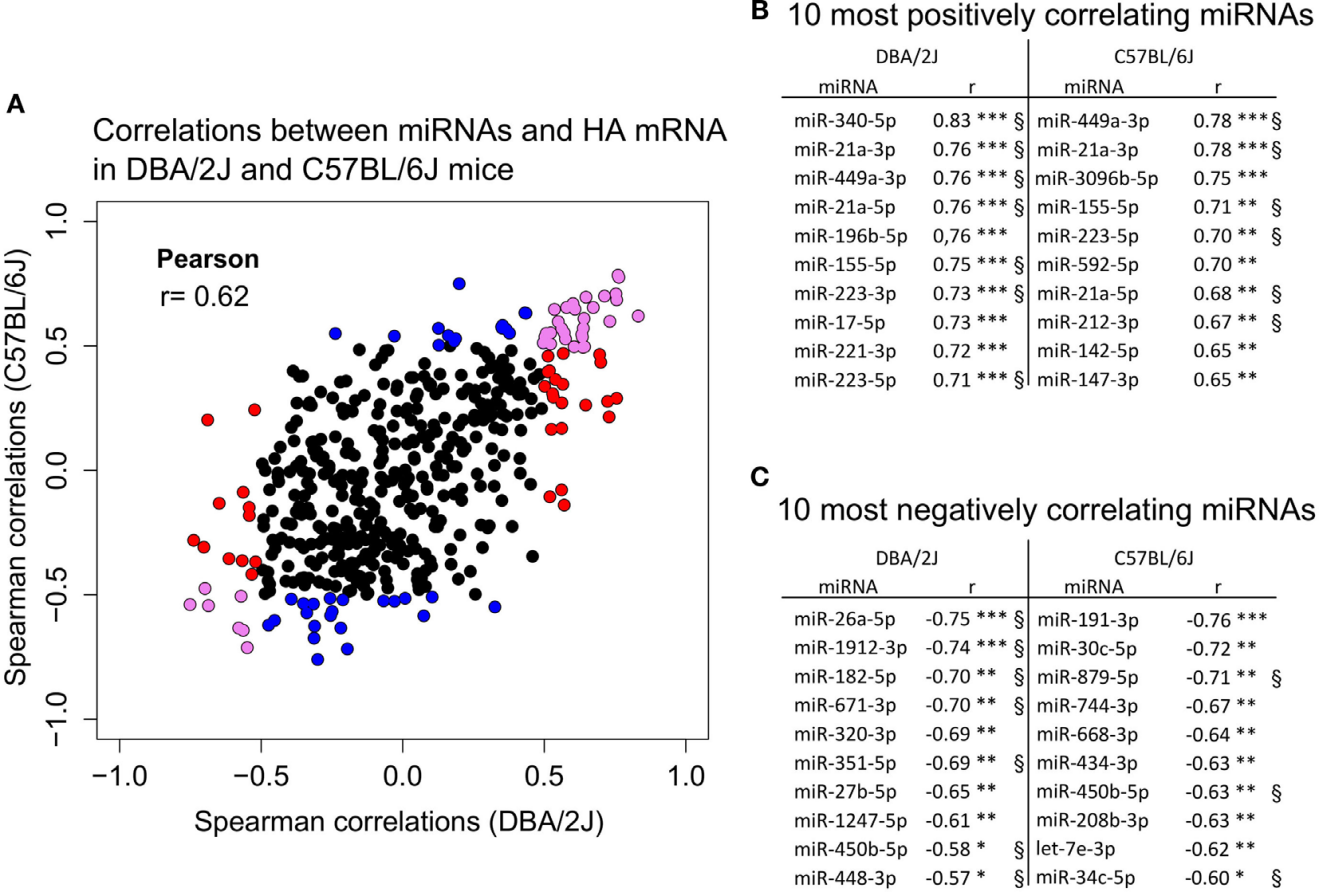
**Correlation of miRNA expression with hemagglutinin (HA) mRNA expression**. Correlation of miRNA expression with HA mRNA expression using small RNA sequencing data of the 473 detected miRNAs (see Materials and Methods) and relative expression of HA mRNA obtained with quantitative real-time PCR (RT-qPCR). For the correlation analysis, cpm values of infected mice minus cpm values of mock-treated control mice were used. **(A)** Correlation of 473 miRNAs with HA mRNA expression in DBA/2J (*x*-axis) and C57BL/6J (*y*-axis) mice. Colors indicate 105 miRNAs that significantly correlated in at least one of the mouse strains. Red dots, miRNAs that correlate in DBA/2J only (ρ ≥ |0.5|, FDR ≤ 0.05); blue dots, miRNAs that correlate in C57BL/6J only (ρ ≥ |0.5|, FDR ≤ 0.05); violet dots, miRNAs whose expression correlates significantly in both mouse strains (FDR ≤ 0.05) with ρ ≥ |0.5| in at least one mouse strain. The Pearson correlation coefficient of 0.62 indicates the correlation of those data between the two mouse strains. **(B)** The 10 miRNAs with the strongest positive correlation with HA mRNA in each mouse strain. **(C)** The 10 miRNAs with the strongest negative correlation with HA mRNA in each mouse strain. ^§^Significantly correlating (ρ ≥ |0.5|, FDR ≤ 0.05) when combining miRNA and HA mRNA expression data of DBA/2J and C57BL/6J mice. These miRNAs, therefore, correlate most strongly in both mouse strains (***p value ≤ 0.001; **p value ≤ 0.01; *p value ≤ 0.05). All *p* values were FDR corrected.

To assess the degree of synchronization between regulation of miRNA populations and viral replication, we then tested to what extent the expression changes of infection-regulated miRNAs correlated with IAV HA mRNA levels. Expression of 105 miRNAs correlated significantly (ρ ≥ |0.5|, *p* ≤ 0.05) with HA mRNA expression in at least one mouse strain (Figure 9), expression of 65 (62%) of which correlated positively. The degree of correlation with HA mRNA expression was similar to some extent between the two mouse strains, showing a significant (*p* ≤ 0.05) linear correlation between DBA/2J and C57BL/6J mice (Pearson correlation = 0.62). Of note, there were no miRNAs that correlated significantly in opposite directions in the two strains. Expression of the eight IAV-regulated miRNAs (except miR-467e-5p) that distinguished between the two mouse strains even before infection (AUC > 0.99) also correlated significantly with HA mRNA levels. Expression of 75 miRNAs, including miRNAs of the miR-21, miR-223, miR-34, and miR-449 correlated with both HA mRNA expression and any of the hematological parameters. Of note, expression of 30 miRNAs correlated with HA mRNA expression in at least one mouse strain, but did not significantly correlate with peripheral blood cell indices. Out of these, miR-208b-3p, miR-467b-5p, miR-345-3p, and miR-744-3p were differentially expressed during infection in both mouse strains. The results suggest that there was substantial, but not perfect, synchronization between miRNome and HA mRNA expression. Accordingly, data shown in Figures 1, 2 and 4 clearly show, for instance, that in the DBA/2J strain HA mRNA expression peaked at 48 hpi, but global miRNA reprogramming continued to evolve through 120 hpi. Taken together, these results suggest that pulmonary miRNA regulation is not driven by viral replication only, but also by aspects of the tissue response, including immune cell infiltration, that can be temporally dissociated from viral replication.

### Stepwise Selection of Putative Functionally Important miRNAs

**Figure 10:**
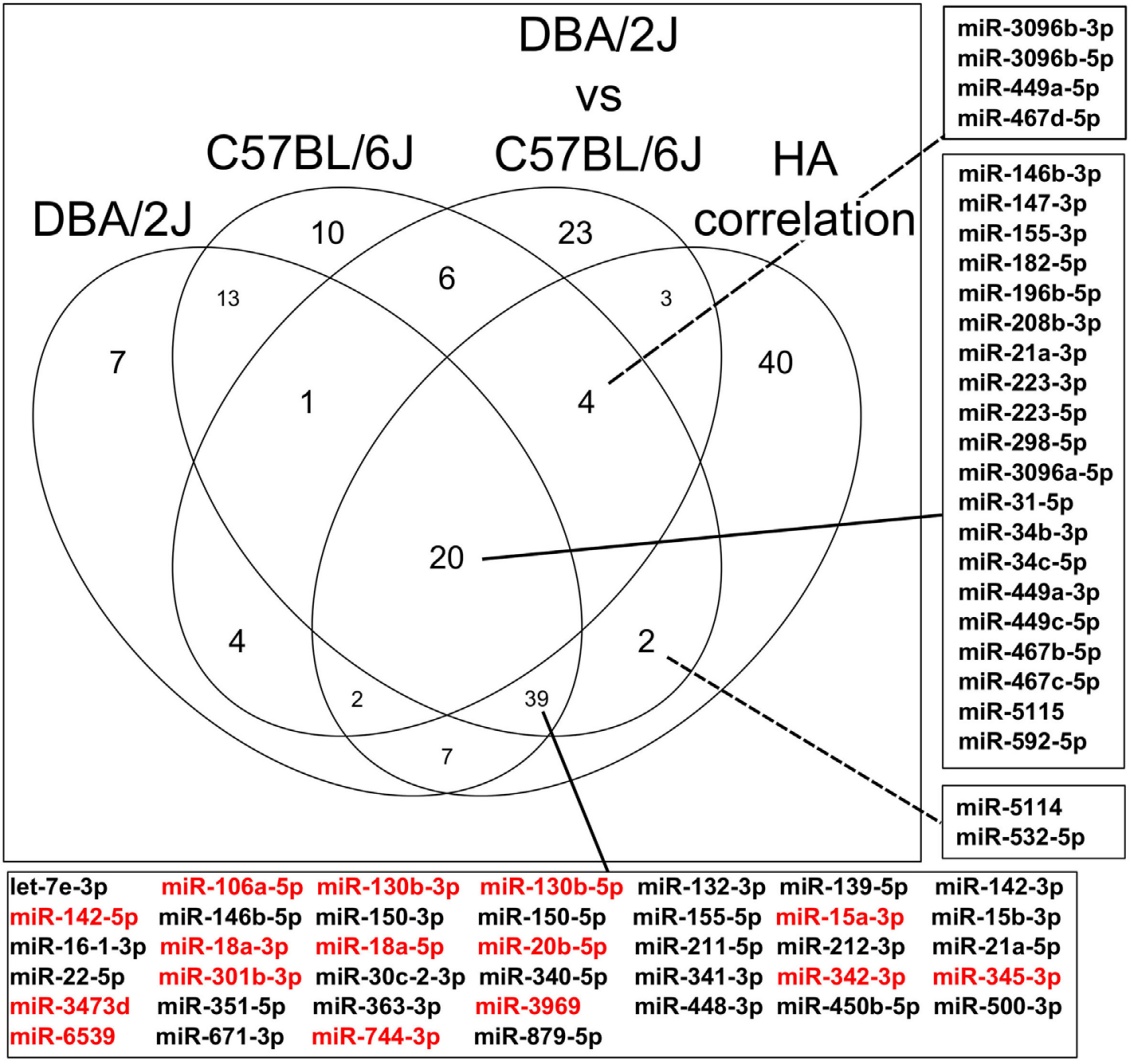
**Venn diagram illustrating sequential identification of subpopulations of miRNAs associated with influenza A virus infection dependent and independent of host genetic background**. The Venn diagram shows the number of miRNAs that are regulated after infection in DBA/2J or C57BL/6J mice, those that are differentially expressed between both mouse strains at any time point (DBA/2J vs C57BL/6J), and those that correlate significantly with viral hemagglutinin (HA) mRNA (HA correlation) expression (see Materials and Methods). The diagram is based on the data shown in Figures 4, 5 and 9. The numbers of miRNAs whose expression differs depending on the host strain include also those miRNAs whose expression does not change during infection. The center box on the right identifies the 20 miRNAs that satisfy all these conditions. The box at the bottom indicates miRNAs that fit all conditions, except that their expression did not differ between the two host strains. Expression of the miRNAs marked in red was particularly similar between the two host strains, having a FC ≤|1.5| and FDR adjusted *p* value ≥ 0.1 (see Materials and Methods). The top and bottom boxes on the right indicate miRNAs that are differentially expressed after infection in C57BL/6J mice only and whose expression correlates with HA mRNA expression.

The Venn diagram (Figure 10) illustrates the stepwise selection of the presumably functionally most meaningful infection-regulated miRNAs. The input consisted of all 473 miRNAs that passed the initial pre-analytical filter (see Materials and Methods). Criteria for selection were (1) differential expression at any time point after IAV infection (FC ≥|1.5|, FDR ≤0.05) in either mouse strain, (2) significant difference in expression between the two mouse strains (ratio ≥|2|, FDR ≤0.01) at any time point, and (3) significant correlation with HA mRNA expression (ρ ≥|0.5|, FDR ≤0.05). Ninety-three miRNAs were regulated in the DBA/2J strain, 95 in the C57BL/6J strain, and 63 were differentially expressed between the two strains at any point in the time course (label “DBA2/J vs C57BL/6J” in Figure 10). Thirty-nine miRNAs were commonly regulated after infection and correlated with HA mRNA expression, indicating that they represent the general miRNome response independent of the host genetic background (box below the diagram). Differential expression of 15 of these miRNAs (indicated in red) compared to uninfected mice was particularly similar between the two strains (FDR >0.1 and FC ≤|1.5|). Expression of 20 miRNAs that were regulated after infection in both strains and correlated with HA mRNA expression in addition differed significantly between the two mouse strains (Figure 10, center intersect and right box). These 20 miRNAs (which included miR34 families, which are strongly associated with regulation of apoptosis and the PI3k-Akt pathway [e.g., Figure 6]) thus constituted part of a highly regulated response that can predominate in either strain, depending on the time after infection, and is likely to play a role in host susceptibility. Expression of six miRNAs (identified in the top and bottom boxes, right side of the figure) was specifically regulated in the C57BL/6J strain and correlated with HA mRNA expression. This suggests that they constitute part of the host response specific to this strain that is most closely associated with IAV replication and may contribute to the relative resistance of this strain to IAV infection.

## Discussion

We have performed a comprehensive comparison of global pulmonary miRNA reprogramming during IAV infection in two inbred mouse strains with genetically determined differential susceptibility to this virus. This analysis revealed common and distinct features in the global miRNA expression pattern, and specific differences even in uninfected lungs, suggesting that host strain resistance/susceptibility may be programmed, at least in part, by the abundance of several miRNAs in the uninfected state.

### Kinetics of miRNome Reprogramming and Role of Infiltrating Immune Cells

In both mouse strains, substantial miRNome reprogramming became evident only by 48 hpi, i.e., well after completion of the first viral replication cycle. In contrast, in a related study using the same mouse strains, we detected infection-driven mRNA expression changes as early as 12 hpi (21). This suggests that miRNome reprogramming occurs with considerable delay compared to mRNA reprogramming and may thus be a reaction to, and not a cause of, mRNA-mediated changes in protein expression. The trajectory analysis suggested that infiltrating cells contribute significantly to pulmonary miRNome reprogramming. In addition, pulmonary abundance of miRNAs with positive correlations with peripheral blood granulocytes and monocyte numbers initially tended to be low. These correlations were most pronounced in the more susceptible DBA/2J strain, and immune cell infiltration of neutrophils and macrophages into the lung on day 2 pi is indeed more extensive in this strain than in C57BL/6J mice (40). These observations suggest a model in which a substantial change in pulmonary miRNA populations is due to immigration of leukocytes into the lung, which likely contribute further to changes in pulmonary miRNomes due to their highly activated phenotype in this intensely infected tissue. Indeed, consistent with reports in IAV in other species (54, 55), several of the differentially expressed miRNAs detected in our study are expressed in immune cells, including in peripheral blood. Of these, miR-223-3p (whose expression correlated the most strongly with granulocyte numbers) is known to play important roles in the differentiation of myeloid cells, including neutrophils (56). By the same token, miRNAs highly abundant in uninfected lungs would stem from resident lung cells, and their abundance would appear to be lower due to “dilution” by the miRNAs expressed by immigrating cells. Obviously, this is a simplified model, as some of the upregulation of the miRNAs with initially low expression can also be due to the response of resident cells, for instance due to the activation of pathways relating to tissue injury or intrinsic immunity. Of note, the major features of miRNA reprogramming observed here agree well with our previous analysis of changes in hematological parameters during IAV infection, using the same mice (25). Similar to miRNA expression, peripheral blood indices (particularly granulocyte numbers) differed between the two strains already before infection, and changes became fully manifest from 48 hpi onward. Thus, pre-programmed differences in leukocyte number and responsiveness likely contribute to the observed differences in pulmonary miRNA populations.

### The General Pulmonary miRNA Response to IAV Infection

The Venn diagram (Figure 10) identifies 39 miRNAs whose expression levels were similar in the two host strains, changed significantly during IAV infection in both strains, and correlated with HA mRNA in either strain. Expression of 15 of these was particularly similar in the two strains (Figure 10, marked in red). Thus, these miRNAs likely constitute a major part of the general miRNA response to IAV infection in mouse lung irrespective of host genetic background. Functionally, this group can be linked to general responses expected in an infected organ. For instance, miR-142-5p plays critical roles in lymphocyte development and homeostasis (57), and miR-106a-5p, miR-130-3p, miR-20b-5p, miR-345-3p, and the miR-15 cluster have been associated with immune or stress responses (58–62). Of note, one of these miRNAs (miR-18a-5p) (as well as miR-223-3p, whose expression in addition differed between the two mouse strains) is among three miRNAs that were recently identified as being commonly regulated in the response to IAV infection in all four species screened, i.e., humans, pig, chicken, and mouse (63). This suggests that part of this general pulmonary response is conserved across evolutionarily diverse hosts of IAV.

### Potential Roles of miRNAs in Host-Strain-Specific Susceptibility and Resistance to IAV Infection

How might differential expression and regulation of miRNAs affect susceptibility in the two strains used? It is not easy to answer this question, as any miRNA may have several mRNAs targets, more than one miRNA can regulate the same mRNA, and both up- and downregulation of miRNAs may significantly affect levels of a given protein. In addition, the DBA/2J strain is considered a more “inflammogenic” host (20, 40) and it is, therefore, possible that a good portion of the miRNome response in this strain relates to the inflammatory response and not antiviral immunity *per se*.

The expression of 20 miRNAs with differential expression between the two host strains correlated significantly with HA mRNA expression, suggesting that they closely relate to host susceptibility or resistance (Figure 10, center). The expression of miRNAs that play a role in host-strain susceptibility or resistance is expected to correlate with the virulence of the pathogen. Indeed, changes in expression of several of these 20 miRNAs (miR-147-3p, miR-155-3p, miR-223-3p, as well as the miR-34 and miR-449 families) correlate with IAV virulence (14, 15, 17, 64). In addition, several of them have been implicated functionally in the host response to IAV (14, 16–18, 64) or other viruses, suggesting that their differential expression in our model is functionally relevant. For instance, miR-449-5p exerts anti-IAV activities by inhibiting histone deacetylase and, therefore, inducing IFNβ expression (18), and inhibition of miR-223-3p (which was more highly expressed in the DBA/2J strain in our study) reduced mortality and delayed death of H5N2-infected mice (64). The miR-34 and miR-449 families control epithelial barrier repair (65) and regulate multiciliogenesis *via* the Delta/Notch pathway (66, 67), which might help transport virions out of the respiratory tract (68) and reduce end-organ damage. Of these two, the miR-449 family is of considerable interest with respect to the higher resistance of C57BL/6J mice, because—in addition to their different abundance in the two strains—these miRNAs were more strongly induced in C57BL/6J mice, suggesting that their higher abundance is less due to mere leukocyte infiltration. Differential expression of several of these 20 miRNAs may affect the outcome of IAV infection by regulating overall inflammation. miR-147-3p has been reported to negatively regulate TLR signaling in murine macrophages (69). miR-223-3p dampens NLRP3 inflammasome activity (70) and can inhibit inflammation by targeting STAT3 (71). However, it was more highly upregulated in the DBA/2J strain, suggesting that its anti-inflammatory effects did not dominate. Two of the 20 miRNAs (miR-31-5p and miR-182-5p) can be linked to adaptive immunity, i.e., T cell activation and regulation of Treg differentiation, respectively (72, 73). However, taken together the results suggest that immune functions of this central group of miRNAs predominantly affect host susceptibility to IAV infection by modulating innate immune responses and inflammation.

### Potential Roles of miRNA Regulation of Apoptosis and the PI3K Pathway in Host Susceptibility

The higher abundance of potentially antiapoptotic miRNAs in the DBA/2J strain suggests a plausible role in the higher susceptibility of this strain. The IAV NS1 protein can activate the PI3K pathway, which leads to antiapoptotic responses and, subsequently, higher virulence (74). The higher abundance of antiapoptotic miRNAs even before infection in DBA/2J mice, therefore, supports the hypothesis that they contribute to host susceptibility by enhancing viral spread. In agreement with this model, several of the miRNAs with differential abundance in the two mouse strains have been shown to regulate the PI3K pathway, which relates strongly to apoptosis (see above) as well as phagocytosis (51, 75). In particular, a subgroup of seven downregulated and abundant miRNAs that were less abundant in DBA/2J mice were enriched for this pathway. The NS1 protein activates the PI3K pathway by binding the SH3 domain of PI3K, and overexpression of heterologous SH3 domain has been shown to inhibit viral replication (76). In our study, putative mRNA targets were enriched for SH3 domain binding proteins, and one may speculate that inhibiting expression of endogenous SH3-binding proteins might have a similar effect by making SH3 domains more accessible to the NS protein. Indeed, induction of *Cxcl10* requires the PI3K/Akt pathway (77), and *Cxcl10* is much more strongly upregulated in DBA/2J than in C57BL/6J mice (20, 21).

### Highly Discriminatory (Predictive) miRNAs

Of note, we identified a group of regulated miRNAs whose abundances differentiated between the two mouse strains before infection and in the earliest stages of infection (Figure 7) and might, therefore, be used to predict the outcome of the infection in the uninfected host. The miR-467 family was strongly represented in this group. In addition to its role in apoptosis, miR-467b-5p targets the Lpl gene in macrophages and thereby limits lipid accumulation and pro-inflammatory cytokine secretion (78). Also, miR-147-3p can dampen TLR-signaling in murine macrophages (69). Thus, their likely function in the host response to IAV is to limit excessive inflammation. For the other miRNAs in this group, reports of functions relating to infection could not be found. However, they might play yet unknown roles and might be interesting candidates for future investigations regarding host-strain susceptibility to IAV infection. Interestingly, miR-3096a-5p and miR-3096b-3p, which by far showed the strongest host-strain differences, may constitute non-canonical (DICER independent) miRNAs (www.mirBase.org).

### miRNAs with Potential Binding Sites in the IAV Genome

The identification of miRNAs whose human homologs have potential binding sites in the IAV genome is of considerable interest. These miRNAs were more highly expressed in C57BL/6J than DBA/2J mice and might, therefore, contribute to the higher resistance of the C57BL/6J strain. Of note, miR-31-5p, miR-379-5p, miR-7a-5p, as well as some members of the miR-449 (-5p) and miR-34 (-5p) families were moderately to highly abundant (>10 CPM), making it more likely that they would bind to a biologically relevant number of viral RNAs.

### miRNAs and Their Counterparts

For some host strain-dependently regulated miRNAs, such as miR-21a-3p and miR-155-3p, we found a high number of validated functions for their counterparts miR-21a-5p and miR-155-5p. We propose that the ratio between these forms might also be relevant to their function. For example, a study found opposite roles of miR-155-3p and miR-155-5p, in that they activated and inhibited TLR7-mediated type I interferon production, respectively. In this model, predominance of miR-155-3p would favor activation of this process.

### Conclusion

Using two mouse strains of greatly differing genetic susceptibility to IAV infection, we found substantial differences in pulmonary miRNA expression even before infection, which evolved further throughout infection and could in part be attributed to differences in leukocyte populations before and during infection. Before infection, host susceptibility was particularly associated with consistently higher expression of miR-208b-3p, miR-3096b-3p, and the miR-467 family and lower expression of miR-3096a-5p and the potentially anti-inflammatory miR-147-3p. During infection, susceptibility correlated with a shift toward higher expression of miRNAs inhibiting apoptosis but lower expression of miRNAs associated with regulation of PI3K-Akt signaling, i.e., two processes known to play important roles in the host response to IAV infection. These results suggest that pulmonary miRNA populations before and during IAV infection are in part determined genetically and contribute to susceptibility to IAV infection in this murine host, and likely in humans.

## Author Contributions

MP: experimental design, animal work, laboratory analysis, data analysis, graphics, and preparation of manuscript. KS: experimental design and preparation of manuscript. FP: initial concept of the study, experimental design, preparation of the manuscript, and supervision of the study.

## Conflict of Interest Statement

The authors declare that the research was conducted in the absence of any commercial or financial relationships that could be construed as a potential conflict of interest.
